# The impact of electronic prescribing systems on pharmacists’ time and workflow: protocol for a time-and-motion study in English NHS hospitals

**DOI:** 10.1136/bmjopen-2015-008785

**Published:** 2015-10-26

**Authors:** Behnaz Schofield, Kathrin Cresswel, Johanna Westbrook, Ann Slee, Alan Girling, Sonal Shah, Jamie Coleman, Aziz Sheikh

**Affiliations:** 1 Centre of Medical Informatics, Usher Institute of Population Health Sciences and Informatics, The University of Edinburgh, Medical School, The Old Medical School, Edinburgh, UK; 2 Faculty of Medicine and Health Sciences, Australian Institute of Health Innovation, Macquarie University, Sydney, New South Wales, Australia; 3 Department of Strategic Systems and Technology, Patients and Information, NHS England, London, UK; 4 School of Health and Population Sciences, The Learning Centre, The University of Birmingham, Birmingham, UK; 5 School of Clinical and Experimental Medicine, College of Medical and Dental Sciences, University of Birmingham, Birmingham, UK; 6 University Hospitals Birmingham NHS Foundation Trust, School of Clinical and Experimental Medicine, College of Medical and Dental Sciences, University of Birmingham, Birmingham, UK

**Keywords:** Time-and-motion study, electronic Prescribing, pharmacists

## Abstract

**Introduction:**

Electronic prescribing (ePrescribing) systems are rapidly being introduced into National Health Systems (NHS) hospitals in England following their widespread earlier adoption into primary care settings. Such systems require substantial changes in the way pharmacists organise their work and perform their roles. There is however as yet limited evidence on the extent to which these changes may support or compromise efficient and safe working practices by pharmacists. Identifying and quantifying these changes, and their effects, is central to informing system and work practice design, as well as informing training and implementation processes. This protocol describes a study to measure the impact of ePrescribing systems on pharmacists’ time and workflow.

**Methods and analysis:**

A direct observational controlled pre–post implementation time-and-motion study will be conducted in six wards at one NHS Trust over two observational periods. Pharmacists will be shadowed and details of all work tasks performed will be collected and time-stamped. Task distribution, frequency and duration will be measured and changes in these measures preimplementation and postimplementation, and between control and intervention wards will be measured. Interviews with pharmacists will investigate their perceptions of the impact of the ePrescribing systems on their work and will be conducted in both periods. The extent to which pharmacists’ expectations of the impact of the ePrescribing systems on their work with postimplementation reports will be qualitatively explored, as will any differences between perceptions and results from the time-and-motion analysis.

**Ethics and dissemination:**

Institutional research ethics approval has been obtained from The University of Edinburgh. Local approval from the participating NHS Trust and informed consent from participating pharmacists have been obtained, while also complying with local governance requirements. The results of the study will be presented at conferences, published in peer-reviewed journals, and shared with members of our Patient and Public Involvement Group, to facilitate wider dissemination.

## Introduction

Electronic prescribing (ePrescribing) systems are now well established in UK primary care,^1^ ^2^ but widespread adoption of fully computerised prescribing systems has to date only been achieved in a few UK hospitals.^3^ The National Health Service (NHS) Connecting for Health has defined ePrescribing as: “The utilisation of electronic systems to facilitate and enhance the communication of a prescription or medicine order, aiding the choice, administration and supply of a medicine through knowledge and decision support and providing a robust audit trail for the entire medicines use process.”

The adpotion of an ePrescribing system is a disruptive innovation that can lead to major workflow changes that are expected to result in a range of benefits, including time savings for professionals. However, existing evidence of work efficiency benefits of ePrescribing when compared to paper-based systems is mixed and differs depending on the health professional groups studied.^4–14^ Studies indicate prescribers spend less time calculating drug dosages and looking for paper charts,^4^ ^5^ and a reduction of pharmacist time spent filling prescriptions and prescription monitoring.^6^ ^7^ However, other evidence indicates that ePrescribing can make some tasks more time-consuming for certain healthcare professionals.^5^ ^8–14^ For example, research has shown increases in time spent on order entry for prescribers.^8^ ^9^ There is also evidence that medicines administration may become more time-consuming.^15^

In light of this mixed evidence base, and the fact that most of the previous work has focused on doctors using bespoke and extensively customised ePrescribing systems in North American settings,^5^ ^7–11^ we seek to investigate the impact of ePrescribing systems on pharmacists’ time and workflows in hospitals in England. This work is important, as investments in and implementation of such systems tend to be based on (among other things) assumptions surrounding time-savings for individual users.^5^ ^16^ This may in turn lead to inflated expectations among users. Concerns regarding changes in work practices has been identified as the central concern of both clinicians and managers in relation to the introduction of ePrescribing in hospitals.^17^ If workflows are disrupted in unexpected ways, increases in workloads and increased time required for certain tasks not anticipated and planned for in advance, then adoption and user satisfaction will be negatively affected.^18^ More realistic expectations are also likely to facilitate training approaches, increase acceptance of systems by healthcare professionals and decrease the risk that the technology is rejected or used in ways other than intended.^19–21^ Pharmacists have been the least studied healthcare professional group in this respect yet the implementation of such electronic systems require substantial changes in the way hospital pharmacists organise their work and perform their role.

As part of a national programme of research being undertaken to inform deliberations on the safe, effective and efficient procurement and implementation of ePrescribing systems into NHS hospitals in England,^22^ we are planning to undertake a rigorous quantitative assessment of the impact of commercially available ePrescribing systems implemented in two English NHS hospitals on pharmacists’ time and workflows and simultaneously qualitatively study pharmacists perceptions and experiences of this transition to ePrescribing.

## Study aims and objectives

The overall aim is to assess the impact of commercially available ePrescribing systems implemented in two hospitals, in one English NHS Trust, on pharmacists’ time and workflows.

Our objectives are to measure time spent undertaking medication related tasks, clinical and non-clinical tasks and communication patterns before and following the implementation of an ePrescribing system at the study hospital sites, and to understand pharmacists’ perceptions and experiences of this move to ePrescribing systems.

## Study design

A direct observational time-and-motion study will be conducted using a controlled before-and-after design with contemporaneous controls. This will take place in a total of six wards at two hospitals in one English NHS Trust over two observational periods. In the intervention wards, observations will be made both before and after the implementation of the ePrescribing system, with contemporaneous observations in the control wards. The participating hospitals will select the study wards based on local plans to implement ePrescribing systems. The intervention wards will not have the ePrescribing system implemented until the end of the first observation period. The control wards will not have the ePrescribing system implemented at any time during the observation periods.

To explore the perceptions of pharmacists of the impact of the system on time and workflows, we will conduct interviews with the pharmacists participating in the time-and-motion study both before and after the implementation of ePrescribing.

## Outcome measures

The primary outcome will be changes (before and after, and between intervention and control groups) in the proportions of time spent on each particular group of tasks as a percentage of the overall time observed. The group of tasks of interest are:

- Time spent with patients.
- Time spent on clinical activities.

Secondary outcomes will include the distribution of the numbers and durations of tasks within each group of tasks.

The tasks observed are presented in online supplementary appendix I.

## Study setting

The two hospitals from which the wards will be selected are ready to implement ePrescribing and have a local requirement for an impact assessment of the new ePrescribing system. In each hospital data will be collected in three wards with similar patient profiles. Two of these wards (one in each hospital) are scheduled to implement the ePrescribing system between the two data collection points. The four remaining wards will function as controls. In these, implementation is scheduled to occur after the conclusion of the study.

We aim to observe individual pharmacists during their working day in comparable wards with relatively high rates of medication-related activity, in order to produce comparable results and capture a range of relevant tasks.

## Participant selection and enrolment

The aim is to observe pharmacists working on each of the control and intervention wards in each hospital. All pharmacists at the participating hospitals, on duty on the wards being observed at the time of the observations, will be invited and will be asked to provide written informed consent to participate. Pharmacists will be invited to participate with the help of relevant pharmacy managers.

At the time of recruitment, pharmacists will also be invited to participate in short semistructured interviews focusing on exploring their perceptions on the impact of ePrescribing on their work. We acknowledge, that the availability of pharmacists at the time of the work will depend on local workloads and staffing levels.

## Ethics, governance and consent

Observations will not include recording of any patient-related data. The research team will supply an information sheet to each participating pharmacist on invitation to participate in observations/interviews. Written consent to take part in observations/interviews will be sought, comprising a signed consent form signed by both researcher and participant. All participants will be encouraged to discuss any questions with the research team prior to data collection. All fieldwork will be undertaken with due regard to maintaining the best interests of participants.

Pharmacists being observed will be asked to verbally notify patients to the presence of the researcher observing the workflow of the pharmacist. If a patient objects, the observer will take a note and cease observations while the pharmacist attends to that patient. To ensure that patients who are not able to provide their assent to the presence of the researcher are not approached, ward managers will be asked to identify these patients in advance. These may include people who are not able to understand the instructions for cognitive or sensory reasons, or lack of English, or who are too distressed or ill to be involved, or who are under 16 years of age. The observer will make a note using a proforma which records these patients’ bed number (not their name). This process will be discussed with ward managers prior to any observations being carried out.

## Data collection

The Work Observation Method by Activity Timing (WOMBAT) tool will be used to collect data.^23^ This allows a set of study-determined task categories and subcategories to be developed. It is based on previous international work carried out by others and will therefore allow for future comparison. For this study we will structure the WOMBAT data collection under four task dimensions of: (1) What (the task being observed); (2) Where (the location where the observed task is being undertaken); (3) With (the person/people with the pharmacist at the time the observed task is being undertaken); and (4) How (how the task is being completed, eg, using a computer). The software also has the capacity to record information about interruptions and multitasking during observations. It will be customised via a web application for the purposes of the present work, and will be installed on tablet computers for data collection by research staff.

Two researchers will be trained to use the tool and inter-rater reliability testing will be conducted to ensure consistency in its application between observers. An initial training period of 4 weeks on all wards at the hospital sites will offer opportunities for refining the task classification and use of the tool by the observers.

A trained observer will follow one pharmacist at a time and record/time predefined tasks over several weeks at different times of the day, according to a defined observational schedule (see below). Each pharmacist will be observed over a number of 2 h periods. The postimplementation data collection will take place approximately 3–6 months after the implementation of the ePrescribing system, in order to allow users to get used to the new system. Wherever possible, the suggested time-and-motion procedures proposed by Zheng *et al*^24^ will be adhered to, to ensure consistency and quality. Identification details of individual participants, wards and hospitals will be kept confidential and the data will be transferred via a web application to a statistical package.

Interviews, guided by topic guides (see online supplementary appendix II), will explore interviewees’ perceptions associated with ePrescribing and work tasks. This will allow comparison of quantitative measurements and pharmacists’ perceptions. Participants will be asked broadly similar questions, but the interviews will be tailored to individual's areas of work and task categories recorded. It is also likely that the topic guide will continue to evolve during the course of the study as more data are gathered. At least 10 interviews will be conducted which should allow for variability of responses and data saturation (the point at which no new information or themes are observed in the data). All interviews will be digitally recorded, subject to participant consent and, together with any accompanying field notes, professionally transcribed verbatim. We expect each interview to last approximately 10–20 min.

## Observation schedule

Each pre/post observation period will be conducted over 4 weeks. Observation sessions of individual ward-based clinical pharmacists will be 2 h long. Two observers will each perform up to 6 h of observations per day. The observers will alternate between the wards on a daily rotation, and will aim to observe pharmacists on all wards at each hospital on any 1 day. The pharmacists will be observed during the allocated 2 h period that may include time on the ward, in the pharmacy department or dispensary.

## Sample size calculations

A total of 410 h (=2×205 h) of observations have been scheduled. This equates to 205 total hours of observation preimplementation and a further 205 h of observations postimplementation. Of the total of 410 observable hours, 130 h will be for observations in the two intervention wards and 280 h in the four control wards. The precision of the effect estimate will depend on the number of individual tasks that go to make up the total time spent on a task group, and on the SD of the length of an individual task. This means, for example, that greater statistical power would be generated if the task group consisted of a large number of short tasks of relatively constant length than if it comprised a smaller number of tasks of more variable length. In the absence of adequate pilot data on the length and variability of individual tasks, calculations under several different assumptions are presented in table 1. The detectable change in the time spent on a particular task varies with the coefficient of variation (CV) (CV=SD/mean) of task duration and the number of relevant tasks per hour.

**Table 1:** Change in task duration (%) detectable with 80% power, assuming 320 h of observations divided equally between intervention and control wards in a controlled before-and-after design

CV of task duration	Number of tasks per hour (for given activity)					
	5	10	15	20	25	30
0.5	13.3	9.4	7.7	6.6	5.9	5.4
1.0	26.6	18.8	15.3	13.3	11.9	10.9
1.5	39.9	28.1	23.0	19.9	17.8	16.3
2.0	53.2	37.5	30.7	26.6	23.8	21.7

CV, coefficient of variation.

For example, if the pharmacist performs, on average, 10 tasks per hour in the ‘time spent with patients’ group of tasks, and these tasks are such that the SD of task duration=mean task duration (ie, CV=1), then the study is powered to detect a change of plus or minus 18.8% in ‘time spent with patients’ time as a result of ePrescribing. These particular assumptions (CV=1, tasks per hour=10) are consistent with the earlier study of doctors’ and nurses’ time spent on medication-related activities, but are not guaranteed to hold here.^13^

## Data analysis

For each group of tasks a difference-in-difference approach will be used. For the main analysis, the effect of the intervention will be estimated as the difference between proportions of time spent on specific task types before and after implementation in the intervention wards, net of the difference in proportions observed over the same period in the control wards. Normal approximations will be used, with p=0.05 as a threshold for statistical significance. This approach has been used by Westbrook *et al*.^13^

Qualitative data collection and analysis will be iterative, allowing emerging themes to be explored further and disconfirming evidence to be sought. Thematic analysis will consist of comparing data within individuals, system functionalities and perspectives. We expect the coding framework to be based on the interview topic guide.

Qualitative and quantitative data will be integrated after each round of data collection (ie, before and after implementation) in order to explore perceptions of changes and time with objective measurements. This will be followed by integrating findings and exploring changes to measurements and perceptions before and after implementation. Two data coders will analyse the data. Data will be analysed within individual hospital sites initially, before making comparisons across sites.^25^

## Discussion

In order to assess the impact of commercially available ePrescribing systems implemented in hospitals on pharmacists’ time and workflows, a direct observational controlled pre–post implementation time and motion study is being conducted. Additionally pharmacists’ perceptions and experiences of this transition to ePrescribing are being explored by conducting personal interviews. The study represents the only time and motion study of hospital pharmacists with ePrescribing as the intervention of interest. As such it provides a useful baseline for future studies. However, the results will relate to two English NHS Trust potentially limiting generalisability. Additionally pharmacists observed may have changed their behaviour as a result of being observed.
